# Multi-Scale Superpixel-Guided Structural Profiles for Hyperspectral Image Classification

**DOI:** 10.3390/s22218502

**Published:** 2022-11-04

**Authors:** Nanlan Wang, Xiaoyong Zeng, Yanjun Duan, Bin Deng, Yan Mo, Zhuojun Xie, Puhong Duan

**Affiliations:** 1 School of Computer and Electrical Engineering, Hunan University of Arts and Science, Changde 415000, China; 2 Center for International Education, Philippine Christian University, Manila 1004, Philippines; 3 School of Electrical and Information Engineering, Changsha University of Science and Technology, Changsha 410114, China; 4 International College, Chongqing University of Posts and Telecommunications, Chongqing 400065, China; 5 College of Electrical and Information Engineering, Hunan University, Changsha 410082, China

**Keywords:** hyperspectral image, superpixel segmentation, structural profiles, image classification, unsupervised feature selection

## Abstract

Hyperspectral image classification has received a lot of attention in the remote sensing field. However, most classification methods require a large number of training samples to obtain satisfactory performance. In real applications, it is difficult for users to label sufficient samples. To overcome this problem, in this work, a novel multi-scale superpixel-guided structural profile method is proposed for the classification of hyperspectral images. First, the spectral number (of the original image) is reduced with an averaging fusion method. Then, multi-scale structural profiles are extracted with the help of the superpixel segmentation method. Finally, the extracted multi-scale structural profiles are fused with an unsupervised feature selection method followed by a spectral classifier to obtain classification results. Experiments on several hyperspectral datasets verify that the proposed method can produce outstanding classification effects in the case of limited samples compared to other advanced classification methods. The classification accuracies obtained by the proposed method on the Salinas dataset are increased by 43.25%, 31.34%, and 46.82% in terms of overall accuracy (OA), average accuracy (AA), and Kappa coefficient compared to recently proposed deep learning methods.

## 1. Introduction

A hyperspectral imager can simultaneously record abundant spectral information and spatial information regarding the observed materials to form a hyperspectral image (HSI). A HSI, which has hundreds of continuous spectral channels for each pixel, can offer very detailed spectral information. Due to this characteristic, a HSI has the unique ability to identify different land covers and has been widely applied in various fields, such as object detection [1,2,3,4], image interpretation [5,6,7], and water quality retrieval [8,9,10]. In recent years, hyperspectral image classification, which aims to assign a unique label to each pixel, has received more attention due to its importance in precision agriculture, urban investigation, and so on.

In the past few decades, a larger number of hyperspectral image classification methods have been developed [11,12,13,14]. The earliest classification methods were based on spectral classifiers, including support vector machine (SVM) [11], random forest (RF) [12], and nearest neighbor (NN) [13]. These methods mainly used spectral information for classification without considering the spatial information of each pixel, which easily produces salt and pepper noise in the classification map [15].

To fully utilize the spatial information in HSI, many spectral–spatial classification methods have been investigated. For example, Benediktsson et al. proposed extended morphological profiles based on the repeated employment of opening and closing morphological operations with increasing sizes [16]. Kang et al. proposed an edge-preserving filtering method for extracting spectral–spatial features of HSIs, in which the spectral number of the input is first reduced, and then, the domain transform recursive filtering is used to remove the image details [17]. Ghamisi et al. developed extinction filters to extract spatial and contextual information about HSIs [18]. Duan et al. developed a structural profile for the feature extraction of HSIs to greatly improve the discrimination of different ground objects, in which the HSI is modeled as a structural component that reflects the significant spatial structures and a texture component that is the image noise and useless details [19]. In addition, in order to increase the classification performances of these feature extractors, several classification frameworks are used, such as multi-scale classification methods [6], multi-view classification methods [7], semi-supervised classification methods [20], and active learning methods [21].

In recent years, deep learning models have been widely applied in the classification of HSIs. For instance, Chen et al. proposed a convolutional neural network (CNN) for the classification of HSIs, where several convolutional and pooling layers are used to extract deep spectral–spatial features [22]. Hang et al. developed cascaded recurrent neural networks (RNN) to learn the discriminative features of HSIs, in which the first RNN is used to remove redundant information of adjacent spectral channels, and the second RNN models the complementary information of nonadjacent spectral channels [23]. Hang et al. designed a multitask generative adversarial network for the classification of HSIs with limited samples, in which a generator network aims to classify the class of the input data, and a discriminator network is used to discriminate the real and fake samples [24]. These methods can achieve satisfactory classification performances when the number of training samples is sufficient. However, it is hard to label a large number of samples by users in real applications. Therefore, some improved classification technologies have been proposed, such as a semi-supervised classification framework [25], ensemble learning [26], and active learning [27].

To improve the classification performance in the case of limited samples, various classification frameworks, including semi-supervised classification, active learning, and multi-scale or multi-view classifications, have been developed [20,28,29,30]. For example, Kang et al. designed a decision function to expand training samples, in which two feature extractors were used to yield initial probability maps [20]. Lu et al. proposed a multi-view kernel collaborative subspace clustering method, in which the subspace learning and collaborative representation were employed to construct the Laplacian matrix [30]. Although these methods can improve the classification performance to some extent, the rich spatial feature and homogeneity of the same object, which are both very important components for obtaining superior classification results with limited samples, have not been further analyzed in hyperspectral images.

Recently, structural profiles have received more attention in the hyperspectral image classification community [6,19,31,32]. For example, Shah et al. used structural profiles in the preprocessing stage to remove texture details, greatly improving the classification performance of the classifier [31]. Liang et al. developed a multi-view structural profile to characterize complex spectral–spatial features of hyperspectral images [32]. These approaches have proven that the structural profile can well represent the intrinsic properties of the input while removing unrelated information. However, they fail to consider the internal homogeneity among adjacent pixels, which easily yields the over-smoothed phenomenon, especially in the case of limited samples. Inspired by the fact that superpixel segmentation can divide an image into several local regions without intersecting, this work proposes a novel multi-scale superpixel-guided structural profile method for hyperspectral image classification. First, the spectral number of hyperspectral images is reduced with the averaging fusion method. Second, the multi-scale structural profiles are constructed with the help of the multi-scale superpixel segmentation technique. Finally, the multi-scale structural profiles are merged to obtain the discriminative features followed by a spectral classifier to yield the final classification map. Experiments on several hyperspectral datasets demonstrate that the proposed method has a superior classification performance over other advanced classification approaches in the case of limited samples. The novelty and contribution of the proposed method are as follows:

(1) This paper presents a novel multi-scale superpixel-guided structural profile classification framework, which can well preserve the boundaries of different land covers.
(2) A novel superpixel-level structural feature is proposed to make full use of the strong correlation between neighborhood pixels, which can provide spectral homogenous information belonging to the same object.
(3) The proposed method does not require much parameter adjusting. The parameters of the proposed method are set to fixed values, which are able to obtain superior classification results over other classification methods in the case of limited samples.

## 2. Proposed Method

**Figure 1:**
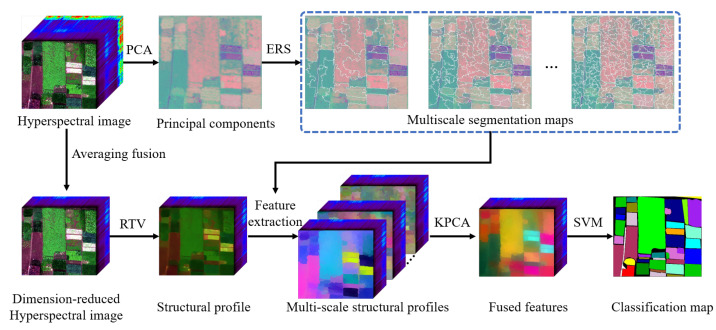
Schematic of the proposed classification method.

Figure 1 shows the schematic of the proposed classification method, which mainly consists of three steps: dimension reduction, multi-scale feature extraction, and feature fusion. First, the dimension reduction technique is used to decrease the spectral dimension. Second, the multi-scale feature extraction aims to provide a better characterization of different objects with different sizes. Finally, the extracted multi-scale structural profiles are integrated with the KPCA method, and the fused features are fed into a spectral classifier to yield the classification map.

### 2.1. Dimension Reduction

In order to decrease the computing cost in the following process, the spectral number of the original image is reduced with an averaging fusion method. In more detail, the *N*-dimensional hyperspectral image I is first segmented into *R* groups of subsets along the spectral direction. Then, the averaging strategy is utilized to merge the spectral bands in each subset to obtain the dimension-reduced hyperspectral data Y.

### 2.2. Extraction of Multi-Scale Structural Profiles

To better characterize the spectral–spatial information of HSIs, we propose multi-scale structural profiles by using the superpixel segmentation technique to preserve the boundaries of different land covers. First, an entropy rate superpixel (ERS) segmentation technique [33] is conducted on the principal components of the input data to produce the homogeneous region. Specifically, the PCA algorithm is performed on the input data I to obtain the first three principal components as the base image $I_{PCs}$ for superpixel segmentation. Then, a graph G=(V,E) is built based on the base image, where *V* represents the vertex set of pixels in the base image, and *E* denotes the edge set that measures the pairwise similarities between neighboring pixels. Next, graph *G* is segmented into *L*-connected subgraphs by choosing a subset of edges A⊆E. To form the homogeneous and compact clusters, the objective function of the ERS segmentation method is constructed by an entropy rate term H(·) and a balance term B(·) as follows:

(1) $$\max_{A}\left\{H\left(A\right)+\lambda B\left(A\right)\right\}\;s.t.\;A\subseteq E$$

where λ denotes the weight for adjusting the contribution of H(·) and B(·). A greedy optimization scheme [34] is adopted to solve the objective function in (1). Finally, the superpixel map is generated by performing the ERS method on the base image $I_{PCs}$ to yield the 2D superpixel segmentation map. In this work, we refer to the ERS segmentation method with ERS as follows:

(2) $$E=\mathrm{ERS}(I_{PCs},T)$$

where E is the segmentation result and *T* denotes the amount of superpixels, which plays an important role in real applications. How to set an optimal value for the amount of superpixels is a very challenging issue since there is no single segmentation region that can accurately characterize the spatial information [35]. When the number of superpixels is relatively small, each superpixel contains more pixels, which causes ambiguity-labeled boundary superpixels that need to be further segmented. When the number of superpixels is relatively large, each superpixel cannot provide distinctive spatial information to infer accurate labels. To alleviate the above-mentioned problem, a multi-scale segmentation strategy is proposed to select the value of *T*. In more detail, the base image is segmented into 2C+1 scales. The amount of superpixels for the *c*th scale is set as $T_{c}$:

(3) $$T_{c}={\left(\sqrt{2}\right)}^{c}T_{f},\;c=0,\pm 1,\pm 2,\cdots ,\pm C$$

where $T_{f}$ denotes an empirical value as a base superpixel number. Considering that the value of $T_{c}$ may not be the integer number, $T_{c}$ is set as $\min(\max\left(1,round\left(T_{c}\right)\right),P)$, where *P* is the total number of pixels for $Y_{PCs}$. Accordingly, the amount of multi-scale superpixels *T* is set to be $\left\{T_{1},T_{2},\cdots ,T_{C}\right\}$.

Then, a relative total variation (RTV) method [36] is conducted on the dimension-reduced hyperspectral data Y to obtain structural profile S.

(4) $$\arg\;\min_{S}\sum_{p=1}^{P}{\left(S_{p}-Y_{p}\right)}^{2}+\alpha \cdot \left(\frac{D_{x}\left(p\right)}{L_{x}\left(p\right)+\varepsilon }+\frac{D_{y}\left(p\right)}{L_{y}\left(p\right)+\varepsilon }\right),$$

where S is the structural profile. α represents a weight parameter. ε denotes a very small positive number to prevent the division by zero. $D_{x}$ and $D_{y}$ are the variations in *x* and *y* directions.

$$D_{x}\left(p\right)=\sum_{q\in R(p)}g_{p,q}\cdot \left|{\left(\partial_{x}S\right)}_{q}\right|$$

(5) $$D_{y}\left(p\right)=\sum_{q\in R(p)}g_{i,j}\cdot \left|{\left(\partial_{y}S\right)}_{q}\right|$$

where $\partial_{x}S$ and $\partial_{y}S$ are the partial derivatives, which aim to estimate the spatial similarity in a local region R(p), and $g_{p,q}$ is defined as:

(6) $$g_{p,q}=\exp\left(-\frac{{\left(x_{p}-x_{q}\right)}^{2}+{\left(y_{p}-y_{q}\right)}^{2}}{2\sigma^{2}}\right).$$

where σ is the standard deviation in the Gaussian filter.

Finally, we use the position indices of pixels for each superpixel $E_{i}$ on the structural profile S to obtain multi-scale 3D superpixels and a mean filtering is performed on each 3D superpixel to obtain the multi-scale structural profiles $S_{c}$ (c=1,2,⋯,2C+1) so as to boost the similarity of pixels belonging to the same object. Specifically, the mean value of pixels in each 3D superpixel is computed. Then, all pixels in each superpixel are assigned with the mean value. Using this operation, the interferences in each superpixel are greatly decreased, which effectively reduces spectral variability belonging to the same object.

### 2.3. Fusion of Multi-Scale Features

The multi-scale structural profiles $\hat{S}$ have very high dimensions and embody plenty of redundant information. This easily leads to the Hugh phenomenon, which is not conducive to the classification of HSIs. Furthermore, the spectral difference of the same object is decreased in $S_{c}$. However, the spectral difference belonging to different ground objects is still small and needs to be further improved. To overcome this problem, the KPCA method [37] is adopted to merge different scales of structural profiles to reduce the redundant information and enlarge the spectral differences of different objects. It should be mentioned that the spatial sizes of all structural profiles are consistent. More specifically, first, different scales of structural profiles are stacked together to obtain multi-scale structural profiles $\hat{S}=\left\{S_{1},S_{2},\cdots ,S_{2C+1}\right\}$. Then, the multi-scale structural profiles $\hat{S}$ are mapped into a high-dimensional feature space *H* with a radial basis kernel function Φ. Finally, the principal components are calculated as:

(7) $$G\alpha =\lambda \alpha \;s.t.\;{∥\alpha ∥}_{2}=\frac{1}{\lambda }$$

where G indicates the Gram matrix $\Phi^{T}\left(\hat{S}\right)\Phi \left(\hat{S}\right)$. In this work, we refer to the KPCA with $\mathrm{KPCA}(\hat{S},K)$, where *K* is the number of the preserved features. Accordingly, the obtained features F are fed into the spectral classifier SVM to obtain the classification result O (Algorithm 1).
**Algorithm 1:** Multi-scale superpixels-guided structural profiles for hyperspectral image classification**Input:**
 Input hyperspectral image I;
**Output:**
 Classification result O;
1:**Begin**2:The spectral number of the input hyperspectral image I is reduced with an averaging-based fusion method to obtain dimension-reduced data Y.3:According to (2), compute the multi-scale superpixel segmentation maps E.4:According to (4), compute the structural profile S.5:Using the position indices of pixels for each superpixel in *c*th superpixel map $E_{c}$ on the structural profile S to obtain multi-scale 3D superpixels.6:Using mean filtering on each 3D superpixel to obtain the multi-scale structural profiles $\hat{S}$.7:According to (7), fuse the multi-scale structural profiles $\hat{S}$ to obtain fused features F.8:The fused features F are fed into a spectral classifier SVM to obtain classification result O.9:**Return**O10:**End**


## 3. Experimental Results

### 3.1. Experimental Setup

(1) Compared methods: To verify the effectiveness of the proposed classification method, several recently proposed or highly cited classification approaches were adopted as compared approaches, including support vector machine (SVM) [11], extended morphological attribute profiles (EMAP) [38], superpixel-based classification via multiple kernels (SCMK) [39], PCA-based edge-preserving features (PCAEPFs) [40], multi-scale total variation (MSTV) [6], orthogonal total variation component analysis (OTVCA) [41], local correntropy matrix (LCEM) [42], spectral–spatial transformer network (SSTN) [43], and spectral–spatial feature tokenization transformer (SSFTT) [44]. For all compared approaches, the default parameters in the corresponding publications were used. The SVM classifier was implemented using the LIBSVM library [45] by using the radial basis function kernel, in which the Gaussian kernel with five-fold cross-validation was adopted for the classifier.
(2) Datasets: In this work, three hyperspectral datasets were employed to test the classification abilities of all considered approaches. These datasets are available from the public classification database.

**Figure 2:**
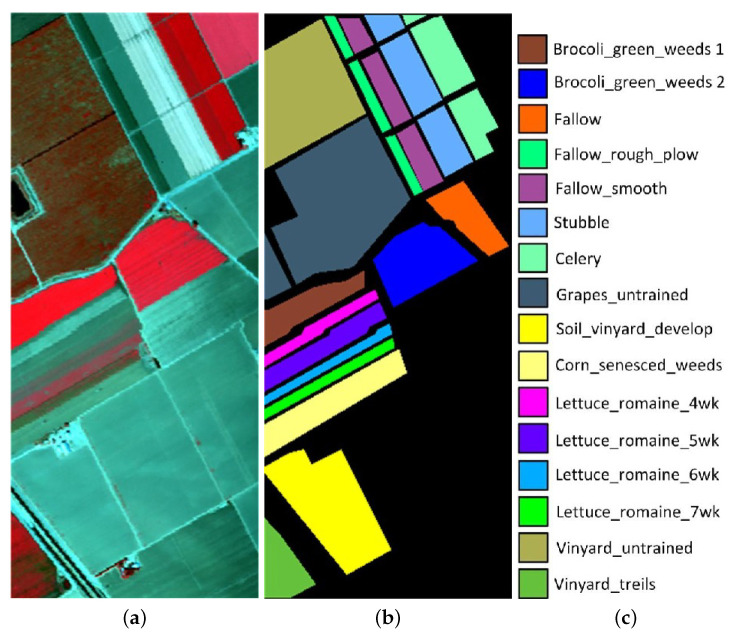
Salinas dataset. (**a**) False color composite. (**b**) Reference image. (**c**) Label name.

The Salinas Pines dataset was obtained by the AVIRIS sensor over Salinas Valley, USA. The dataset contained 224 spectral bands ranging from 0.4 to 2.5 μm. The spatial resolution was 3.7 m and its spatial size was 512 × 217. Before the classification experiment, 20 water absorption spectral channels (no. 108–112, 154–167, and 224) were removed. Figure 2 presents the false-color image and the ground truth.

**Figure 3:**
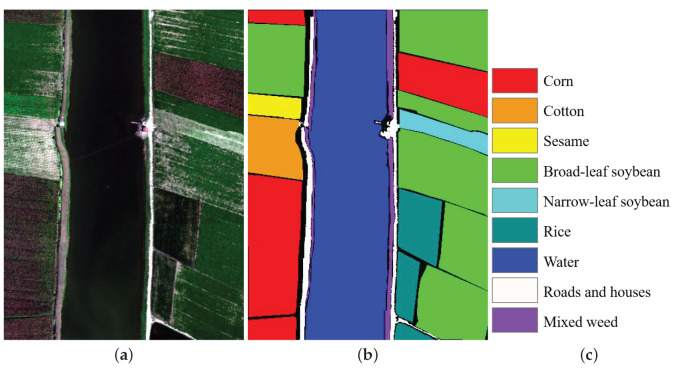
Longkou dataset. (**a**) False color composite. (**b**) Reference image. (**c**) Label name.

The Longkou dataset was obtained by an 8-mm focal length Headwall Nano-Hyperspec imaging sensor, over Longkou, Hubei province, China, which was captured by a DJI Matrice 600 Pro UAV platform (DJI, Shenzhen, China). The flying height of the UAV was 500 m. The spatial size of the dataset contained 550 × 400 pixels, and it consisted of 270 spectral bands ranging from 0.4 to 1 μm. The spatial resolution of this dataset was about 0.463 m. Figure 3 presents the false-color image and the reference image.

**Figure 4:**
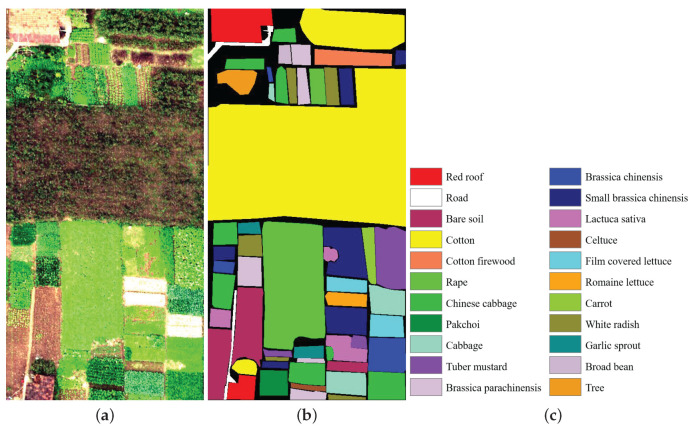
Honghu dataset. (**a**) False color composite. (**b**) Reference image. (**c**) Label name.

The Honghu dataset was obtained by a 17-mm focal length Headwall Nano-Hyperspec imaging sensor embedding on a DJI Matrice 600 Pro UAV platform over Honghu City, Hubei province, China. This study region is a complex agricultural scene, which contains different types of crops and different cultivars of the same crop are shown in the area, such as *Brassica chinensis* and small *Brassica chinensis*. The flying height of the UAV was 100 m. This image consisted of 270 spectral bands ranging from 0.4 to 1 μm. The spatial size was 940 × 475 pixels with a spatial resolution of 0.043 m. Figure 4 shows the false-color image and the reference image.

(3) Objective indices: To objectively evaluate the classification performances of all used methods, three extensively used classification indices [46,47,48] were adopted, i.e., overall accuracy (OA), average accuracy (AA), and Kappa coefficient. For these objective indices, a higher value indicates a better classification performance.

### 3.2. Classification Performance

#### 3.2.1. Salinas Dataset

**Figure 5:**
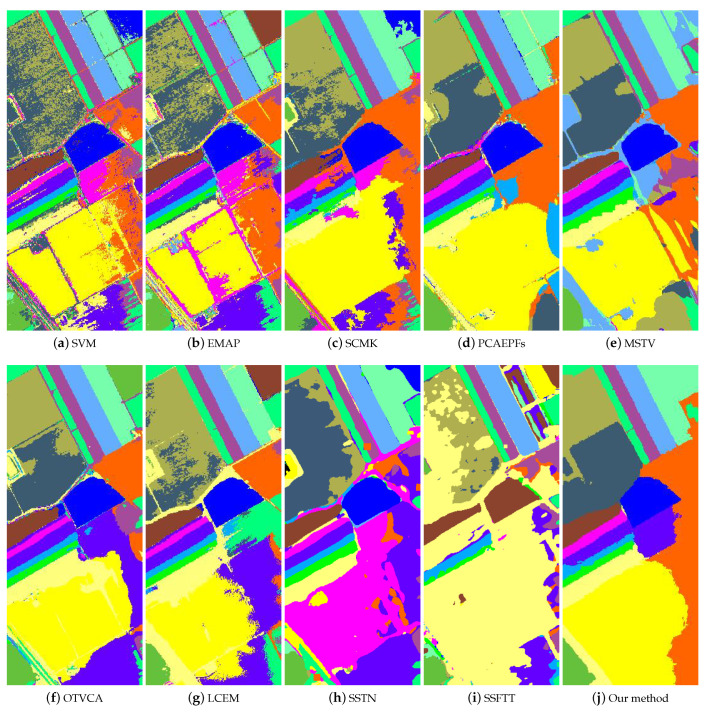
Classification maps of different classification methods on the Salinas dataset. (**a**) SVM [11], OA = 80.08%. (**b**) EMAP [38], OA = 85.54%. (**c**) SCMK [39], OA = 88.70%. (**d**) PCAEPFs [40], OA = 95.12%. (**e**) MSTV [6], OA = 94.46%. (**f**) OTVCA [41], OA = 93.04%. (**g**) LCEM [42], OA = 90.15%. (**h**) SSTN, OA= 69.67%, (**i**) SSFTT, OA= 39.93%, (**j**) Our method, OA = 98.05%.

The first experiment was tested on the Salinas dataset. To examine the classification performances of different classification methods in the case of limited samples, the number of training samples per class was 5, which was randomly selected from the reference image. Figure 5 presents the classification results of all studied approaches. As depicted in this figure, the SVM method produces a very noisy classification map and some classes have serious misclassification phenomena. The main reason is that the SVM method is a spectral classifier without utilizing the spatial correlation between neighboring pixels. The EMAP method also yields an unsatisfactory classification effect. There are many salt and pepper noises in the classification map since the EMAP method is a pixel-wise feature extraction method. Compared to previous methods mentioned above, the SCMK method improves the classification performance because this method takes the object-level features into consideration by using the superpixel segmentation technique in the feature extraction process. The PCAEPF method greatly removes the noisy spots in the classification map. However, the edges between different ground objects have mislabels. Similar to the PCAEPF method, the MSTV method also produces noisy labels on the boundaries of different objects. The OTVCA method fails to well classify some similar land covers, such as Vinyard_untrained and Grapes_untrained classes. For the LCEM method, the Grapes_untrained class is misclassified into the Vinyard_untrained class. The SSTN and SSFTT methods cannot work well in the case of limited samples and the classification maps have serious misclassification labels. The reason is that the two methods are based on deep learning models, which usually require a larger number of training samples. By contrast, the proposed method obtains the best classification results among all classification approaches in preserving the edges of different land covers.

Furthermore, Table 1 presents the objective results of all classification methods. By observing this table, several conclusions can be obtained. First, the SVM method obtains the lowest classification accuracies in terms of OA, AA, and Kappa coefficient. The reason is that the SVM method only uses the spectral information of each pixel. Second, the pixel-level feature extraction method (e.g., EMAP) is inferior to the spectral–spatial classification method (e.g., SCMK, PCAEPF, MSTV). Third, the deep learning methods based on SSTN and SSFTT produce very low classification accuracies. In addition, some classes could not be identified (the primary reason is that the number of training samples is very limited in this work). Finally, the proposed method obtains the highest OA, AA, and Kappa coefficients among all methods. Moreover, the proposed method yields the highest classification accuracy in six classes. This objective result is consistent with the visual classification map.

#### 3.2.2. Longkou Dataset

**Figure 6:**
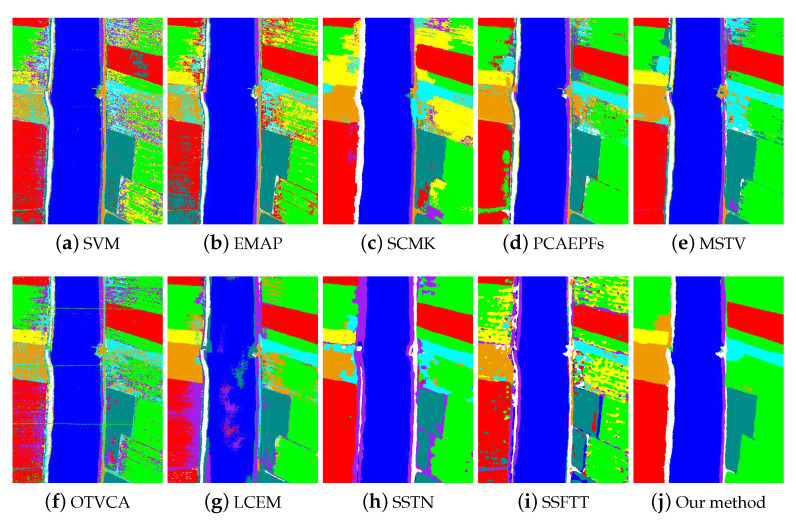
Classification maps of different classification methods on the Longkou dataset. (**a**) SVM [11], OA = 76.47%. (**b**) EMAP [38], OA = 81.20%. (**c**) SCMK [39], OA = 85.53%. (**d**) PCAEPFs [40], OA = 91.36%. (**e**) MSTV [6], OA = 91.51%. (**f**) OTVCA [41], OA = 82.51%. (**g**) LCEM [42], OA = 87.32%. (**h**) SSTN, OA= 90.38%, (**i**) SSFTT, OA= 81.22%, (**j**) Our method, OA = 92.63%.

The second experiment was conducted on the Longkou dataset, where the number of training samples per class was set to 5. Figure 6 presents the visual classification results of all considered classification techniques. As shown in this figure, the SVM method obtained the “ pepper and noisy” classification map because only the spectral reflectance of each pixel was utilized. The classification performance of the EMAP method was improved. However, there were still noisy labels in the homogeneous region. The SCMK method discarded some misclassification labels in the homogeneous regions. Nevertheless, some areas had obvious inaccurate classifications. The PCAEPF method improved the classification effect. However, there were obvious misclassifications in the boundaries of different ground objects. Compared to the PCAEPF method, the MSTV method removed some noisy labels, but it could not well identify the edges of different land covers. The OTVCA method had a poor classification performance. The classification result had very serious misclassifications, such as noisy spots. For the LCEM method, some classes had obvious misclassifications, such as water. The SSFTT method also yielded a very noisy classification map. The SSTN method improved the classification performance. However, there were misclassifications on the edges of different classes. Different from these methods, the proposed method had a satisfactory classification performance. The homogeneous regions could be well preserved in the classification map.

Moreover, Table 2 shows the classification accuracy of all used methods. The recorded values of all methods are the mean of ten experiments. For the SVM method, the classification accuracies were relatively low since the spectral classifier only took the spatial information into consideration. The EMAP and OTVCA methods improved classification accuracies, and they obtained similar classification accuracies. However, the AA value was still low. The SSTN method boosted the classification accuracies over the other compared methods. Generally, among all classification methods, the proposed method obtained the highest classification accuracies in terms of OA, AA, and Kappa. The OA increased from 76.47% in the SVM method to 92.63% obtained by our method. In general, our method greatly improved the classification accuracies compared to other classification techniques in the case of limited samples.

#### 3.2.3. Honghu Dataset

**Figure 7:**
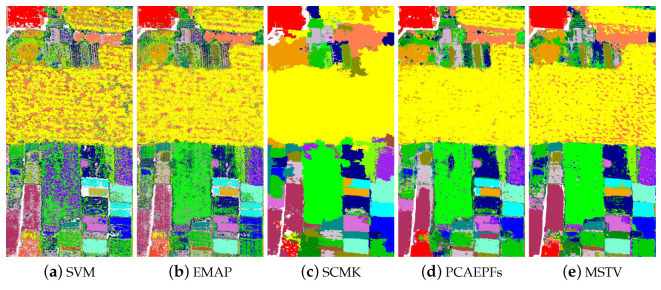

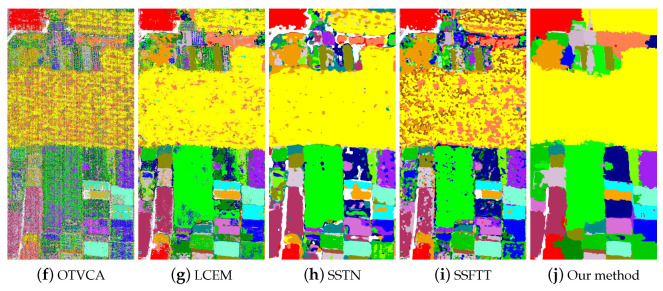
Classification maps of different classification methods on the Honghu dataset. (**a**) SVM [11], OA = 54.21%. (**b**) EMAP [38], OA = 65.37%. (**c**) SCMK [39], OA = 81.17%. (**d**) PCAEPFs [40], OA = 82.69%. (**e**) MSTV [6], OA = 86.25%. (**f**) OTVCA [41], OA = 45.56%. (**g**) LCEM [42], OA = 78.57%. (**h**) SSTN, OA= 81.71%, (**i**) SSFTT, OA= 66.92%, (**j**) Our method, OA = 89.68%.

The third experiment was performed on the Honghu dataset, where the number of training samples was set to 10. Figure 7 shows the classification results of all methods. The SVM method still had a very noisy classification performance in the resulting image. Likewise, the EMAP method also had a similar classification result. Most land covers cannot be well identified. The SCMK method greatly boosted the classification result. However, the edges of different land covers were not aligned with those of real objects. For the PCAEPF method, the classification map had obvious noisy spots. The MSTV method yielded similar phenomena to the PCAEPF method. The OTVCA method cannot classify different types of crops when the number of training samples is limited. This is due to the fact that this technique cannot well distinguish the similar spectra belonging to different land covers. The LCEM method also yielded obvious misclassification in the visual map. The SSTN method obtained a similar classification map to the SCMK method. The SSFTT method still cannot perform well on this dataset. The classification map had serious speckle noise. In contrast, the proposed method can yield a better classification map with respect to other approaches. The noisy labels in the homogeneous regions were well removed. Moreover, the edges of different objects were well aligned with those of the reference image.

Table 3 lists the objective values of different classification methods. Since the Honghu dataset had different cultivars of the same crop, the spectral curves of these land covers were very similar. Thus, many methods cannot produce satisfactory classification accuracies. For example, the OTVCA method on the Honghu dataset yielded very low classification accuracies. In contrast, the proposed method obtained the best classification performance in terms of OA, AA, and Kappa coefficient. When the number of training samples was 10, the OA value obtained by the proposed method reached 89.68%.

## 4. Discussion

### 4.1. The Influences of Different Parameters

**Figure 8:**
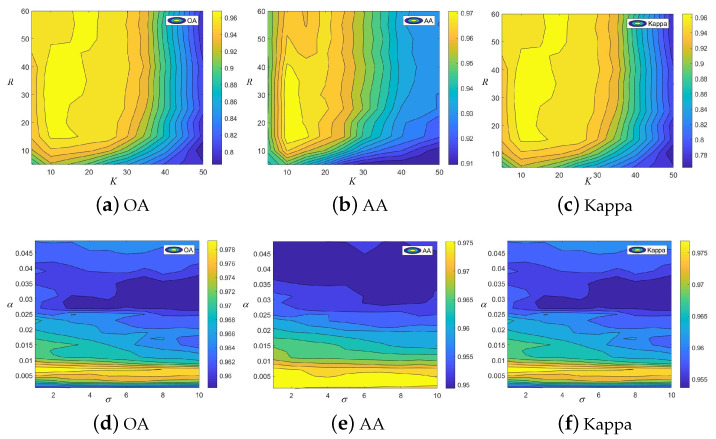
The influences of different free parameters on the classification performance of the proposed method. (**a**) OA obtained by the proposed method with different *R* and *K*. (**b**) AA obtained by the proposed method with different *R* and *K*. (**c**) Kappa obtained by the proposed method with different *R* and *K*. (**d**) OA obtained by the proposed method with different α and σ. (**e**) AA obtained by the proposed method with different α and σ. (**f**) Kappa obtained by the proposed method with different α and σ.

In this subsection, four parameters, i.e., dimension reduced parameter *R*, the number of feature fusion in the KPCA *K*, weight parameter α, and the filter kernel σ, are first discussed. An experiment was conducted on the Salinas dataset, in which the number of training samples was set to 5. Figure 8 presents the influence of four free parameters on the classification performance of the proposed method. When we discuss the influence of the two parameters, i.e., dimension reduced parameter *R*, the number of feature fusion in the KPCA *K*, the weight parameter α, and the filter kernel σ are fixed. The first row in Figure 8 depicts the influence of parameters *R* and *K*. It can be observed that the proposed method can obtain the highest classification accuracies when *R* is 30 and *K* is 15. Similarly, when the parameters α and σ are analyzed, the dimension reduced parameter *R* and the number of feature fusions in the KPCA *K* are fixed. The second row in Figure 8 presents the influence of parameters α and σ. It is obvious that when α and σ are set to 0.007 and 3, respectively, the proposed method has the best classification performance. Therefore, the four free parameters, i.e., *R*, *K*, α, and σ, in the proposed method are set to 30, 15, 0.007, and 3, respectively.

**Figure 9:**
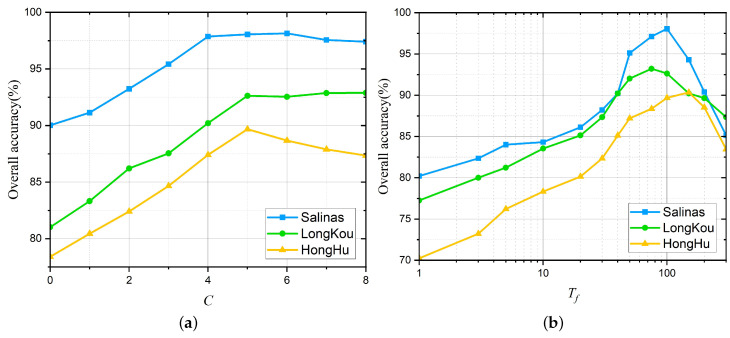
The influence of the number of scales *C* and the number of base superpixel $T_{f}$. (**a**) The number of scales *C*. (**b**) The number of base superpixel $T_{f}$.

Furthermore, the influence of the two parameters, i.e., the number of scales *C*, and the number of base superpixels $T_{f}$, is analyzed. An experiment is performed on the Salinas dataset, where the number of training samples is set to 5. Figure 9a shows the OA obtained by the proposed method as a function of the number of scales *C* whose value is selected from 0 to 8 with step 1. It can be seen that when the number of scales *C* is relatively small, the proposed method produces unsatisfactory classification performance. When *C* is 5, the proposed method obtains the highest OA. Figure 9b presents the influence of the number of base superpixel $T_{f}\in \left\{1,3,5,10,20,30,40,50,75,100,150,200,300\right\}$ to the OA of the proposed method. It is obvious that when the number of base superpixel $T_{f}$ is 100, the proposed method yields satisfactory classification performance. Based on the above experiments, the number of scales *C* and the base superpixel number $T_{f}$ are set to 5 and 100, respectively.

### 4.2. The Influences of Different Steps

In this subsection, the effects of different steps, i.e., dimension reduction, superpixel segmentation, structural profile, and feature fusion, over the classification performance of the proposed method are discussed. An experiment was performed on the Salinas dataset, where the number of training samples was 5 for each class. Table 4 shows the classification accuracies of the proposed method with or without these steps. Here, WDR denotes the proposed method without the dimension reduction step. WMS indicates the proposed method without the multi-scale superpixel segmentation step. WSP is the proposed method without the structural profile extraction step. WFF represents the proposed method without the feature fusion step. As shown in this Table, it can be seen that the WSP method obtains the lowest classification accuracies since this method fails to well characterize the spectral–spatial information of HSI. This also illustrates that the structural profile plays an important role in the proposed method. In addition, the WDR method has the least reduction in classification accuracies, indicating that the dimension reduction step has less influence on the proposed method. In general, the proposed method with these steps produces the best classification performance, which demonstrates that all steps make important contributions to the proposed classification method.

### 4.3. The Influences of Different Numbers of Samples

**Figure 10:**
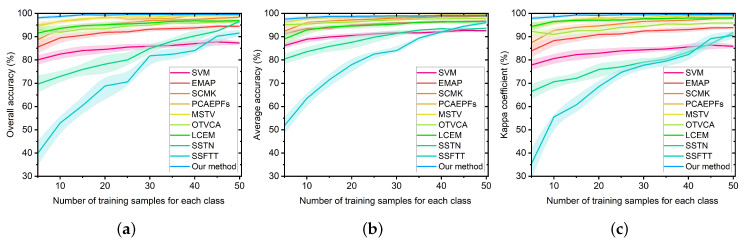
Classification effects of different classification approaches on the Salinas dataset with different amounts of training samples per class (varying from 5 to 50 with step 5). (**a**) Overall accuracy (OA). (**b**) Average accuracy (AA). (**c**) Kappa coefficient. The widths of the line areas indicate the standard deviations of accuracies obtained in ten repeated experiments.

In this subsection, the influences of different numbers of training samples per class on the classification effects of all considered methods are discussed. An experiment was conducted on the Salinas dataset, in which the number of training samples varied from 5 to 50 with step 5. Figure 10 shows the classification accuracies of different classification approaches with different amounts of training sets. From this figure, it can be seen that the proposed method was always higher than other classification approaches. In particular, when the number of training sets was very limited, the proposed method was far more than the other approaches. This also illustrates that the main advantage of the proposed method is that it is able to yield superior classification performance in the case of limited samples. Furthermore, as the number of training samples increases, the classification performances of all classification methods tend to increase. As shown in Figure 10, when the number of training samples was 50, the proposed method produced satisfactory classification performance.

### 4.4. Computing Time

In this subsection, the computing costs of all methods were evaluated. Table 5 shows the computing times of all considered approaches on three hyperspectral datasets. Here, the computing time includes the whole running time of all steps, i.e., training and test times. All experiments were performed on a laptop with 8GB RAM. It can be seen from Table 5 that the computing times of different approaches tend to increase as the spatial size of the input data increases. Moreover, the computing cost of the proposed classification method is acceptable among all methods. For example, the running time of the proposed method on Salinas is only about 18 s. How to improve computational efficiency is an interesting topic.

## 5. Conclusions

In this study, we propose multi-scale superpixel-guided structural profiles for hyperspectral image classification. This method is mainly composed of three key steps. First, the spectral numbers of the input data are decreased with the average fusion method. Second, the multi-scale structural profiles are extracted with the help of the superpixel segmentation technique. Finally, the multi-scale structural profiles are merged with the KPCA method. Experiments on three public datasets reveal that the proposed method can achieve satisfactory classification performances with respect to other classification techniques. The main advantage of this method is that the proposed method is superior to other approaches especially when the number of training samples is very limited.

## Figures and Tables

**Table 1:** Classification accuracies of different classification approaches on the Salinas dataset.

Classes	SVM	EMAP	SCMK	PCAEPFs	MSTV	OTVCA	LCEM	SSTN	SSFTT	Our Method
1	99.13 (0.97)	99.98 (0.05)	97.53 (5.50)	**100.0 (0.00)**	**100.0 (0.00)**	**100.0 (0.00)**	99.49 (1.13)	99.65 (0.21)	86.46 (2.21)	**100.0 (0.00)**
2	98.74 (1.00)	99.75 (0.14)	98.90 (3.48)	99.95 (0.08)	99.97 (0.05)	98.65 (1.24)	97.37 (3.30)	99.70 (0.11)	0	**100.0 (0.00)**
3	79.50 (8.77)	94.55 (2.25)	96.40 (7.65)	97.72 (2.38)	97.87 (2.83)	99.78 (0.16)	**100.0 (0.00)**	34.31 (5.32)	16.09 (6.54)	99.46 (0.10)
4	96.00 (2.41)	95.93 (1.39)	90.01 (8.71)	93.19 (4.02)	96.9 (1.79)	**97.98 (1.38)**	96.47 (1.36)	97.99 (0.15)	96.41 (2.31)	97.13 (0.24)
5	94.01 (7.06)	96.66 (6.27)	97.93 (1.56)	98.98 (3.21)	96.86 (1.49)	98.85 (0.73)	98.65 (1.98)	76.13 (5.48)	86.18 (3.67)	**99.96 (0.00)**
6	99.79 (0.53)	99.41 (0.73)	99.75 (0.00)	99.98 (0.04)	98.34 (2.92)	**99.99 (0.01)**	99.83 (0.45)	99.89 (0.11)	**100.0 (0.00)**	99.97 (0.01)
7	95.27 (3.61)	96.27 (3.16)	99.85 (0.06)	99.92 (0.06)	96.23 (5.52)	**100.0 (0.00)**	99.43 (1.14)	99.41 (0.17)	24.61 (5.49)	99.83 (0.01)
8	64.94 (4.82)	80.00 (7.80)	69.96 (8.23)	93.66 (6.53)	92.73 (5.95)	94.03 (5.53)	87.54 (4.39)	80.37 (2.46)	4.01 (8.75)	**98.15 (2.86)**
9	98.66 (0.87)	98.99 (0.21)	**99.91 (0.19)**	99.58 (0.46)	98.61 (1.03)	99.12 (0.53)	99.04 (1.26)	0	0	99.33 (0.67)
10	77.21 (6.38)	87.98 (4.27)	82.44 (14.88)	**99.31 (0.97)**	95.02 (7.28)	96.28 (1.83)	91.90 (4.40)	92.80 (2.64)	100.0 (0.00)	98.98 (0.20)
11	82.43 (10.35)	79.41 (10.37)	94.93 (5.93)	95.04 (4.63)	**99.65 (0.78)**	95.31 (2.09)	98.22 (2.47)	98.50 (2.13)	0.28 (5.24)	97.00 (7.90)
12	89.11 (7.96)	88.64 (6.36)	91.05 (12.43)	94.71 (4.32)	**99.11 (1.13)**	91.81 (4.11)	94.53 (2.33)	99.48 (0.35)	15.62 (4.65)	98.53 (2.45)
13	79.04 (12.81)	92.32 (3.45)	93.94 (10.28)	91.81 (11.56)	**95.32 (6.68)**	87.78 (12.61)	87.87 (13.79)	98.25 (1.36)	96.51 (2.41)	81.42 (6.59)
14	85.09 (12.77)	**97.23 (1.08)**	88.92 (3.95)	92.54 (10.36)	89.54 (10.17)	89.00 (11.82)	96.92 (3.47)	100.0 (0.00)	70.75 (6.22)	94.78 (6.22)
15	46.93 (5.71)	56.84 (7.75)	82.66 (11.52)	84.52 (8.47)	84.53 (7.06)	74.64 (6.83)	67.34 (8.83)	24.90 (5.48)	54.78 (5.49)	**95.30 (4.80)**
16	93.27 (5.57)	96.43 (2.26)	93.83 (10.51)	99.96 (0.08)	97.68 (4.67)	99.90 (0.11)	96.07 (7.45)	85.88 (4.57)	78.03 (4.67)	**100.0 (0.00)**
OA	80.08 (1.96)	85.54 (2.57)	88.70 (2.84)	95.12 (1.37)	94.46 (1.47)	93.04 (1.70)	90.15 (2.30)	69.67 (3.58)	39.93 (5.10)	**98.05 (0.56)**
AA	86.19 (1.07)	91.27 (1.07)	92.38 (2.16)	96.30 (0.94)	96.14 (1.18)	95.19 (1.26)	89.07 (2.52)	80.45 (2.16)	51.85 (3.59)	**97.49 (0.76)**
Kappa	77.87 (2.15)	83.97 (2.81)	87.47 (3.15)	94.56 (1.53)	93.84 (1.64)	92.27 (1.88)	94.42 (1.58)	66.49 (3.24)	35.52 (6.54)	**97.83 (0.63)**

**Table 2:** Classification accuracies of different classification approaches on the Longkou dataset.

Classes	SVM	EMAP	SCMK	PCAEPFs	MSTV	OTVCA	LCEM	SSTN	SSFTT	Our Method
1	92.11 (5.63)	89.08 (4.71)	94.53 (5.55)	97.17 (2.19)	**98.50 (0.87)**	86.94 (7.66)	94.16 (4.87)	99.30 (1.04)	89.64 (1.65)	97.03 (3.84)
2	43.91 (10.93)	49.86 (11.54)	83.90 (7.02)	64.74 (11.05)	73.27 (6.78)	50.83 (6.96)	57.59 (9.62)	68.25 (3.24)	88.20 (1.42)	**85.49 (14.25)**
3	16.87 (8.07)	16.37 (6.50)	93.82 (13.91)	65.10 (17.34)	66.14 (21.43)	61.68 (17.23)	94.10 (7.59)	96.53 (1.54)	79.47 (2.33)	**97.53 (3.59)**
4	92.60 (6.50)	95.85 (4.61)	67.87 (10.23)	97.47 (2.22)	**98.48 (2.09)**	93.10 (3.55)	98.92 (2.14)	84.88 (3.54)	61.25 (4.62)	96.32 (2.65)
5	23.37 (4.15)	28.49 (7.69)	**94.97 (11.61)**	47.35 (13.52)	36.06 (6.94)	34.08 (8.90)	68.93 (13.45)	92.63 (2.68)	75.18 (3.22)	61.46 (15.93)
6	79.10 (8.70)	76.01 (8.59)	87.65 (2.38)	86.75 (5.51)	89.90 (9.82)	80.61 (9.94)	69.91 (12.19)	89.16 (3.35)	85.11 (1.78)	**97.11 (3.19)**
7	99.94 (0.04)	99.41 (0.33)	99.26 (1.93)	99.92 (0.13)	99.96 (0.09)	**99.98 (0.02)**	98.66 (1.14)	97.33 (1.04)	98.71 (1.02)	99.61 (0.07)
8	59.61 (13.35)	80.77 (14.64)	70.51 (14.0)	**84.62 (9.66)**	80.74 (7.16)	63.55 (14.23)	78.60 (8.97)	43.90 (4.55)	70.49 (2.46)	57.98 (17.46)
9	19.99 (5.77)	**85.83 (10.94)**	69.67 (8.64)	80.99 (11.19)	80.07 (12.46)	34.98 (19.68)	43.29 (17.00)	91.75 (1.77)	43.20 (5.68)	72.50 (17.16)
OA	76.47 (2.98)	81.20 (3.31)	85.53 (3.11)	91.36 (1.63)	91.51 (1.73)	82.51 (4.56)	87.32 (3.19)	90.39 (1.54)	81.22 (2.47)	**92.63 (1.53)**
AA	58.61 (2.13)	69.07 (3.53)	84.69 (3.58)	80.45 (3.46)	80.35 (3.83)	67.31 (5.69)	83.90 (3.89)	84.85 (2.69)	76.81 (3.59)	**85.01 (3.36)**
Kappa	70.47 (3.41)	76.26 (3.94)	81.72 (3.74)	88.87 (2.05)	89.07 (2.16)	77.72 (5.54)	78.24 (3.18)	87.63 (2.51)	76.25 (3.64)	**90.43 (1.94)**

**Table 3:** Classification accuracies of different classification approaches on the Honghu dataset.

Classes	SVM	EMAP	SCMK	PCAEPFs	MSTV	OTVCA	LCEM	SSTN	SSFTT	Our Method
1	88.47 (7.14)	89.31 (6.69)	83.08 (5.59)	91.88 (2.54)	**96.78 (1.84)**	72.22 (15.38)	92.29 (8.87)	83.79 (2.15)	92.53 (1.45)	81.86 (4.73)
2	51.39 (7.73)	55.06 (7.04)	**76.72 (10.98)**	64.93 (7.57)	68.32 (9.38)	30.95 (7.49)	62.33 (11.62)	96.01 (1.05)	91.65 (1.23)	48.72 (16.72)
3	88.14 (11.19)	90.53 (7.55)	83.77 (7.47)	94.04 (3.67)	95.42 (3.58)	71.23 (11.89)	93.05 (4.53)	83.35 (2.60)	62.47 (3.20)	**94.67 (3.80)**
4	96.25 (1.53)	97.42 (0.55)	85.39 (4.39)	99.34 (0.23)	99.36 (0.13)	94.72 (0.63)	98.53 (0.68)	93.21 (1.87)	58.54 (5.63)	**99.54 (0.34)**
5	13.89 (3.56)	17.13 (5.61)	**92.10 (1.72)**	58.56 (14.94)	50.97 (18.12)	7.49 (1.62)	39.57 (13.69)	59.84 (5.41)	55.19 (4.10)	73.69 (8.29)
6	81.06 (5.05)	88.71 (4.04)	86.15 (4.48)	93.09 (4.25)	93.71 (2.03)	71.81 (9.01)	87.32 (4.34)	91.88 (1.58)	86.79 (2.15)	**96.93 (4.74)**
7	61.57 (12.17)	65.12 (8.17)	64.05 (8.78)	79.61 (6.51)	87.48 (3.16)	50.16 (6.48)	**86.17 (3.75)**	52.95 (4.11)	55.84 (3.56)	77.55 (11.02)
8	6.30 (1.56)	9.28 (1.45)	86.79 (9.57)	24.56 (4.93)	31.12 (5.22)	5.96 (1.07)	18.42 (4.24)	7.30 (2.31)	29.43 (4.32)	**90.68 (9.96)**
9	92.17 (6.74)	94.04 (5.37)	92.14 (4.66)	95.12 (5.18)	97.32 (2.21)	82.08 (8.56)	**98.55 (0.95)**	89.28 (2.41)	96.88 (1.04)	97.26 (3.78)
10	27.99 (10.24)	42.26 (14.59)	65.71 (5.86)	68.38 (12.43)	78.43 (6.80)	26.17 (7.89)	69.60 (8.06)	70.23 (3.01)	79.08 (2.78)	**82.31 (15.01)**
11	24.32 (5.70)	35.05 (8.59)	61.2 (8.28)	64.58 (11.79)	64.02 (6.78)	19.45 (5.74)	50.25 (8.12)	60.72 (4.05)	47.34 (5.68)	**78.10 (15.55)**
12	32.72 (5.62)	37.12 (5.90)	78.87 (9.92)	52.18 (9.59)	54.52 (9.76)	19.64 (1.93)	44.79 (6.53)	44.48 (3.05)	70.03 (2.44)	**87.39 (5.47)**
13	44.79 (10.98)	53.56 (7.55)	60.4 (5.17)	70.05 (14.09)	74.31 (6.67)	31.32 (7.21)	54.60 (12.46)	53.01 (3.64)	55.40 (3.02)	**79.47 (10.71)**
14	45.58 (12.39)	40.87 (8.13)	77.96 (12.48)	72.4 (10.09)	**82.64 (9.82)**	30.65 (11.88)	80.70 (8.31)	74.63 (2.58)	79.22 (2.68)	75.67 (6.96)
15	4.33 (1.32)	18.42 (10.84)	**96.65 (1.36)**	54.03 (11.49)	77.88 (13.77)	2.13 (0.59)	60.72 (18.92)	93.81 (1.22)	91.91 (1.67)	84.79 (8.81)
16	77.39 (9.23)	79.65 (7.68)	88.63 (5.82)	97.64 (1.84)	96.85 (2.45)	77.31 (7.75)	89.78 (2.93)	71.61 (2.41)	91.79 (2.65)	**98.18 (1.21)**
17	46.09 (11.69)	46.16 (14.08)	89.48 (10.10)	74.38 (11.32)	65.00 (17.33)	33.19(6.19)	78.82 (12.43)	68.97 (4.77)	64.75 (1.54)	**94.28 (5.38)**
18	14.13 (4.30)	19.5 (5.99)	**90.48 (6.11)**	45.67 (6.13)	59.11 (7.59)	15.72 (3.08)	26.87 (7.32)	68.07 (4.53)	80.97 (2.15)	70.66 (7.95)
19	42.95 (11.09)	52.92 (7.26)	69.37 (11.37)	66.99 (9.90)	**79.28 (10.11)**	38.90 (9.99)	65.31 (9.81)	93.49 (1.46)	76.91 (2.54)	76.36 (17.97)
20	18.14 (5.58)	44.12 (9.01)	93.87 (3.92)	50.08 (6.69)	60.46 (4.73)	12.31 (2.38)	73.69 (10.50)	96.35 (1.45)	78.68 (3.20)	**83.48 (4.89)**
21	9.37 (1.71)	13.71 (4.54)	**97.16 (8.76)**	32.87 (11.34)	51.99 (16.62)	4.27 (0.52)	20.34 (3.54)	39.16 (5.10)	83.88 (1.02)	93.73 (2.96)
22	14.46 (4.86)	18.74 (6.47)	**95.71 (7.17)**	40.35 (13.54)	69.25 (7.72)	10.65 (2.98)	42.17 (8.53)	94.87 (1.07)	94.82 (0.36)	71.58 (7.43)
OA	54.21 (2.48)	65.37 (4.03)	81.17 (1.72)	82.69 (1.61)	86.25 (1.97)	45.56 (3.65)	78.57 (1.55)	81.71 (2.45)	66.92 (1.20)	**89.68 (1.22)**
AA	44.61 (1.51)	50.39 (1.76)	82.53 (0.83)	67.76 (1.81)	74.28 (2.72)	36.74 (1.37)	73.62 (1.79)	72.14 (3.10)	73.83 (2.08)	**83.49 (1.40)**
Kappa	47.36 (2.32)	58.80 (4.16)	76.95 (1.90)	78.55 (1.82)	82.88 (2.29)	38.19 (3.28)	65.18 (2.39)	77.28 (2.87)	61.82 (3.32)	**87.09 (1.49)**

**Table 4:** Classification accuracies of the proposed method with or without several key steps.

Methods	WDR	WMS	WSP	WFF	Our Method
OA	92.21 (1.24)	90.12 (1.27)	83.35 (2.67)	88.21 (2.34)	98.05 (0.56)
AA	92.84 (1.02)	91.03 (0.95)	89.47 (1.39)	90.51 (2.06)	97.49 (0.76)
Kappa	91.34 (2.37)	89.41 (1.36)	81.48 (2.44)	87.71 (3.12)	97.83 (0.63)

**Table 5:** Computing times of different classification approaches on three hyperspectral datasets (in seconds).

Datasets	SVM	EMAP	SCMK	PCAEPFs	MSTV	OTVCA	LCEM	SSTN	SSFTT	Our Method
Salinas	21.08	4.56	4.23	12.98	12.34	64.06	105.49	29.92	29.80	18.26
Longkou	43.87	15.09	10.62	11.63	26.31	176.77	194.11	71.24	76.73	35.22
Honghu	392.66	197.41	33.81	71.21	105.97	416.7	275.95	294.40	302.21	168.36

## Data Availability

Not applicable.

## Abbreviations

HSI	hyperspectral image
SVM	support vector machine
RF	random forest
NN	nearest neighbor
CNN	convolutional neural network
RNN	recurrent neural network
ERS	entropy rate superpixel
RTV	relative total variation
KPCA	kernel principal component analysis
EMAP	extended morphological attribute profiles
SCMK	superpixel-based classification via multiple kernels
PCAEPFs	PCA-based edge-preserving features
MSTV	multi-scale total variation
OTVCA	orthogonal total variation component analysis
LCEM	local correntropy matrix
SSTN	spectral–spatial transformer network
SSFTT	spectral–spatial feature tokenization transformer
